# Bile acids modulate glucocorticoid metabolism and the hypothalamic–pituitary–adrenal axis in obstructive jaundice^☆^

**DOI:** 10.1016/j.jhep.2009.10.037

**Published:** 2010-05

**Authors:** Alison D. McNeilly, David P. Macfarlane, Emmett O’Flaherty, Dawn E. Livingstone, Tijana Mitić, Kirsty M. McConnell, Scott M. McKenzie, Eleanor Davies, Rebecca M. Reynolds, Helle C. Thiesson, Ole Skøtt, Brian R. Walker, Ruth Andrew

**Affiliations:** 1 Endocrinology Unit, Centre for Cardiovascular Science, Queen’s Medical Research Institute, University of Edinburgh, 47 Little France Crescent, Edinburgh EH16 4TJ, UK; 2 MRC Blood Pressure Group, Glasgow Cardiovascular Research Centre, University of Glasgow,126 University Place, Glasgow G12 8TA, UK; 3 Physiology and Pharmacology, University of Southern Denmark, DK-5000 Odense C, Denmark

**Keywords:** HPA, hypothalamic–pituitary–adrenal, ACTH, adrenocorticotropic hormone, 3αHSD, 3α-hydroxysteroid dehydrogenase, FXR, farnesoid X receptor, *Cyp7a1*, cholesterol 7α-hydroxylase, 11βHSD, 11β-hydroxysteroid dehydrogenase, BDL, bile duct ligation, THB, tetrahydrocorticosterone, DHB, dihydrocorticosterone, GCMS, gas chromatography mass spectrometry, CDCA, chenodeoxycholic acid, CA, cholic acid, DCA, deoxycholic acid, GCDCA, glyco-CDCA, *K*_i_, inhibitor constant, TCDCA, tauro-CDCA, DMEM, Dulbecco’s modified Eagle’s medium, PCR, polymerase chain reaction, FF, fat-free, ECRP, endoscopic retrograde cholangiopancreatography, ANOVA, analysis of variance, ALT, alanine transaminase, ALP, alkaline phosphatase, *Cyp11b1*, 11β-hydroxylase, NEFA, non-esterified fatty acids, SEM, standard error of mean, Bile acid, Glucocorticoid, 5β-reductase, Adrenal, Jaundice

## Abstract

**Background & Aims:**

Suppression of the hypothalamic–pituitary–adrenal axis occurs in cirrhosis and cholestasis and is associated with increased concentrations of bile acids. We investigated whether this was mediated through bile acids acting to impair steroid clearance by inhibiting glucocorticoid metabolism by 5β-reductase.

**Methods:**

The effect of bile acids on glucocorticoid metabolism was studied *in vitro* in hepatic subcellular fractions and hepatoma cells, allowing quantitation of the kinetics and transcript abundance of 5β-reductase. Metabolism was subsequently examined *in vivo* in rats following dietary manipulation or bile duct ligation. Finally, glucocorticoid metabolism was assessed in humans with obstructive jaundice.

**Results:**

In rat hepatic cytosol, chenodeoxycholic acid competitively inhibited 5β-reductase (*K*_i_ 9.19 ± 0.40 μM) and reduced its transcript abundance (in H4iiE cells) and promoter activity (reporter system, HepG2 cells).

In Wistar rats, dietary chenodeoxycholic acid (1% w/w chow) inhibited hepatic 5β-reductase activity, reduced urinary excretion of 3α,5β-tetrahydrocorticosterone and reduced adrenal weight. Conversely, a fat-free diet suppressed bile acid levels and increased hepatic 5β-reductase activity, supplementation of the fat-free diet with CDCA reduced 5β-reductase activity, and urinary 3α,5β-reduced corticosterone. Cholestasis in rats suppressed hepatic 5β-reductase activity and transcript abundance.

In eight women with obstructive jaundice, relative urinary excretion of 3α,5β-tetrahydrocortisol was significantly lower than in healthy controls.

**Conclusion:**

These data suggest a novel role for bile acids in inhibiting hepatic glucocorticoid clearance, of sufficient magnitude to suppress hypothalamic–pituitary–adrenal axis activity. Elevated hepatic bile acids may account for adrenal insufficiency in liver disease.

## Introduction

Activation of the hypothalamic–pituitary–adrenal (HPA) axis and enhanced release of cortisol is crucial to a successful response to stress, but this homeostatic mechanism is disrupted in liver disease. In cirrhosis, impaired responsiveness of the adrenal to ACTH contributes to increased mortality with haemodynamic impairment [1,2]. Replacement with low-dose hydrocortisone significantly improves resolution of shock and survival [3]. Similarly, in cholestatic rats, secretion of corticotrophin-releasing hormone is suppressed, and adrenal responses to stress are impaired [4]. However the cause of dysregulation of the HPA axis is not understood.

If metabolism of cortisol is impaired, then negative feedback control of the HPA axis causes suppression of ACTH levels, atrophy of the adrenal gland and a reduced rate of production of cortisol, a pattern also seen in cirrhosis [5,6]. As the liver is the major site of cortisol metabolism [7], impaired clearance of cortisol in liver disease may be caused by the reduced functional liver mass or by an inhibitor of glucocorticoid metabolism.

Hepatic enzymes that inactivate glucocorticoids include 5α- and 5β-reductases and 3α-hydroxysteroid dehydrogenase (3αHSD), which convert cortisol into tetrahydrometabolites [8]. In addition, 5β-reductase and 3αHSD are involved in bile acid synthesis [9]. Bile acids are cytotoxic, so their formation and elimination are tightly regulated by the up-regulation of genes encoding proteins that induce their detoxification and/or excretion, and by the suppression of genes (mainly through the farnesoid X receptor (FXR)) encoding proteins that regulate cholesterol catabolism, e.g., cholesterol 7α-hydroxylase (*CYP7A1*) [9].

Inhibition of renal 11βHSD2 by bile acids impairs glucocorticoid inactivation [10,11] and contributes to the sodium retention and potassium wasting observed in cirrhosis and cholestasis [12] as well as following bile duct ligation (BDL) [13], through illicit occupation of mineralocorticoid receptors by excess cortisol. Bile acids also inhibit hepatic 11βHSD1 [8,14–16], preventing glucocorticoid reactivation. Effects on glucocorticoid metabolism other than by 11βHSDs have not been investigated, although inhibition of 5β-reduction of aldosterone by bile acids has been demonstrated [17].

We hypothesised that bile acid accumulation in cholestasis inhibits hepatic 5β- reductase, contributing to impaired glucocorticoid clearance and attenuation of HPA axis activity. The effects of bile acids on the activity and transcription of hepatic 5β-reductase were investigated *in vitro* in liver and in hepatoma cells. The effect of bile acids on HPA activity was assessed *in vivo* in rats following dietary manipulation [18] or BDL. Glucocorticoid metabolism was also studied in humans following obstruction of the common bile duct by gallstones.

## Materials and methods

Sources unless stated: solvents (Rathburn, Walkerburn, UK), cell culture reagents (Gibco BRL, Paisley, UK), molecular biology reagents (Promega, Southampton, UK), chemicals (Sigma–Aldrich, Poole, UK), radiochemicals (GE-Healthcare, Aylesbury, UK).

### Effects of bile acids on enzyme kinetics in vitro

All experiments followed the guidelines of the Home Office, UK or the Danish Animal Experiments Inspectorate. Male Wistar rats (9 weeks; Harlan Olac, Bicester, UK) were sacrificed by decapitation (08:00 h) within 60 s of being disturbed.

[^3^H]_4_-Tetrahydrocorticosterone (5β-THB) (5β-reductase activity) was generated in hepatic cytosol (100 μg/ml protein), incubated 4 h with [^3^H]_4_-corticosterone (25 nM), corticosterone (975 nM, IC_50_; 0.01–1000 μM, kinetics) and an NADPH-generating system [19]. Conversion of 5β-dihydrocorticosterone (5β-DHB; 2 μM) to 5β-THB (3αHSD activity) was measured following incubation (10 min) as above. 5β-THB was quantified by gas chromatography mass spectrometry (GCMS) [20].

Bile acids (chenodeoxycholic acid (CDCA), cholic acid (CA), deoxycholic acid (DCA), glyco-CDCA (GCDCA) or tauro-CDCA (TCDCA), (10^–2^–10^–9^M) were added to incubations. Inhibition of velocity (IC_50_) was calculated relative to controls without bile acid. *K*_i_ values were calculated by a global fit model of competitive inhibition (*K*_mapp_ = *K*_m_ * (1 + *I*)/*K*_i_; *Y* = *V*_max_ * *X*/(*K*_mapp_ + *X*) using CDCA at its IC_50_.

### Effect of bile acids on transcript abundance in cultured cells

H4iiE cells (ECACC, UK), were maintained in Dulbecco’s modified Eagle’s medium (DMEM, Invitrogen, UK) supplemented with foetal calf serum (10% v/v), penicillin (100 IU/ml), streptomycin (100 μg/ml), and L-glutamine (2 mM) (37 °C, humidified carbon dioxide:air (5:95)). Cells were transferred to fresh media 1 h prior to the addition of bile acid (100 μM) or vehicle (ethanol, <1% v/v) and then incubated (16 h) in triplicate. Total RNA was isolated using Trizol® Reagent (Invitrogen, UK) and cDNA generated using First-Strand cDNA Synthesis Kit. Quantification of transcripts (normalised to cyclophilin A) was performed by qPCR (PRISM 7900, Applied Biosystems, UK). Primers and probes were either designed using *Primer Express* Software (5β-reductase, 3αHSD) or by Applied Biosystems (*Cyp7a1*, NM012942; cyclophilin A, NM017101).

### Effect of bile acids on promoter activity of 5β-reductase

A fragment of the 5′ untranslated region of h*AKR1D1* (5β-reductase) representing the region –383 to +446 kb (relative to transcription start site) [21] was cloned from RPCI-11 HS BAC clone 386M10 (Invitrogen, UK). A construct was generated using high-fidelity PCR amplification using the following oligonucleotides, incorporating a *Kpn* site (underlined) and confirmatory sequencing performed.

5′AKR1D1 (−383), GGTACCAGTCCTGCTGCATCCAAATC;
3′AKR1D1 (+446) GGTACCTGTGGAGAACCTGACTGTAGGA.

Amplimers were inserted into the multiple cloning site of the promoter-less firefly luciferase reporter vector, pGL3-Basic, and their orientations were confirmed. Maxi DNA preparations of the luciferase construct were prepared using Qiagen maxi kits (Crawley, UK).

The human hepatoblastoma cell line, HepG2 (ATCC, Rockville, USA), was cultured in DMEM supplemented with foetal calf serum, L-glutamine and penicillin as above. Plasmid DNA (∼10 μg) was transfected [22] into cells, along with pCH110 plasmid (2 μg; β-galactosidase (β-Gal), Amersham, UK). CDCA (50 μM) or ethanol was added 24 h after transfection. Luciferase activity was assayed in cell lysates 72 h after transfection [23]. Transfection efficiency was assessed by β-Gal activity assayed using the Tropix Galacto-Light kit (Cambridge Bioscience, UK). Experiments were performed in triplicate three times utilising more than one preparation of plasmid.

### Effects of bile acids *in vivo* in rats

#### Dietary manipulation of bile acids

Male Wistar rats (4–6 weeks; *n* = 8/group) were singly housed (6 days). In the first protocol, animals received standard chow ± CDCA (1% w/w, 4 weeks). In the second, they received a fat-free (FF) diet (D05052506; Research Diets Inc, USA) ± CDCA (1% w/w; D05052507) instead.

Daily production of glucocorticoids was studied in urine from animals housed in metabolic cages for 6 days, after 3 weeks on their respective diets. In the first protocol only, responses to restraint stress were studied after 2.5 weeks of the diet. Animals were acclimatised to handling (7 days) and then placed in restraint tubes (20 min, 08:00) and returned to normal cages. Blood was obtained at 0 (immediately prior to restraint) and at 20, 40, 60, and 90 min following restraint.

#### Bile duct ligation (BDL)

The common bile duct was ligated in male Wistar rats (*n* = 6/group; M&B Ejby, Denmark) or rats subjected to sham surgery. Rats were sacrificed by decapitation after 7 weeks, when jaundice and hepatosplenomegaly were evident and decompensated liver failure had occurred [13].

#### Ex vivo measurements

Enzyme activities were determined at the following substrate concentrations: 5β-reductase (25 nM, 1 μM) and 3αHSD (1 μM). Plasma and hepatic biochemistry and urinary steroids were quantified [19,20,24,25]. Bile acids were quantified using a Total Bile Acid Kit (Trinity Biotech, Ireland) [26], and liver function tests were quantified using a Modular P Analyser (Roche Diagnostics, Switzerland). Transcripts of 5β-reductase and *Cyp11b1* were quantified by qPCR [19,27] and normalised to 18S RNA or β-actin (Applied Biosystems), respectively. Transcript abundance of *Cyp7a1* was quantified by northern blot analysis [24] and normalised to U1 (M14386) [19].

### Cortisol metabolism in obstructive jaundice in humans

With Local Ethical Committee approval (06/S1103/38) and written informed consent, women (*n* = 8; 48.2 ± 7.0 y) were recruited following hospitalisation with obstructive jaundice secondary to gallstone disease and studied prior to endoscopic retrograde cholangiopancreatography (ERCP). Healthy control women (*n* = 5; 40.0 ± 7.4 y) were recruited by advertisement. Subjects receiving systemic corticosteroid therapy within 3 months were excluded. Serum biochemical data were recorded, and an overnight urine collection obtained the night before ERCP and steroids was quantified by GCMS [28].

### Statistical analysis

Results are mean ± SEM. *In vitro* data were analysed by ANOVA with post hoc Fisher LSD tests. *In vivo* data were analysed by unpaired Student’s *t* tests or by one-way or repeated measure ANOVA. Correlations were analysed by Pearson’s product moment and partial correlation analyses.

## Results

### Effects of bile acids on enzyme kinetics *in vitro*

**Fig. 1:**
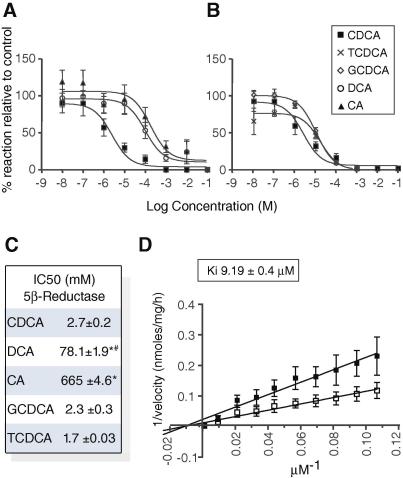
**Inhibition of 5β-reductase by bile acids.** 5β-Reduction of corticosterone in the presence of (A) CDCA, CA, DCA (B) CDCA, GCDCA, TCDCA. Velocity *vs.* Control (100%), without bile acids. (C) IC_50_ of the reactions. (D) Lineweaver–Burke plots showing competitive inhibition of 5β-reductase by CDCA (open squares: 2.5 × 10^–6^ M) (*vs.* vehicle (filled)). Mean ± SEM; *n* = 5. ^∗^*p* <0.05 *vs.* CDCA; ^#^*p* <0.05 DCA *vs.* CA.

5β-Reductase activity was inhibited significantly by bile acids, with an order of potency of CDCA = TCDCA = GCDCA > DCA > CA (Fig. 1A–C). Bile acids and their conjugates did not inhibit 3αHSD (14.29 ± 2.53 Control *vs.* 13.25 ± 3.02 CDCA; 13.87 ± 4.41 TCDCA; 18.44 ± 0.76 GCDCA nmol/mg/h). CDCA was a competitive inhibitor of 5β-reductase (Fig. 1D).

### Effects of bile acids on mRNA levels of metabolising enzymes *in vitro*

**Fig. 2:**
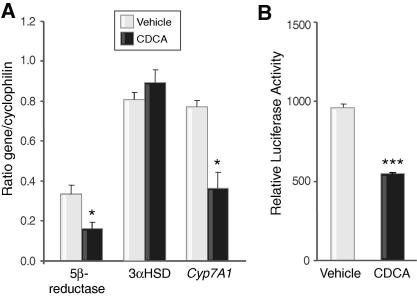
**Effect of CDCA on transcription of steroid-metabolising enzymes.** (A) Abundance of mRNAs of rat 5β-reductase and *Cyp7a1* but not 3αHSD was suppressed in H4iiE cells by CDCA (100 μM), *n* = 5. (B) Activity of the promoter of human 5β-reductase was reduced by CDCA (50 μM). Data are fold induction of luciferase activity relative to control plasmid. (*n* = 3 triplicates), mean ± SEM; ^∗^*p* <0.05, ^∗∗∗^*p* <0.001 *vs.* vehicle.

CDCA reduced the transcript abundance of 5β-reductase (Fig. 2A) and *Cyp7a1* but not that of 3αHSD. CDCA also suppressed the activity of the promoter of 5β-reductase (Fig. 2B).

### Effects of CDCA supplementation in rats on standard chow diet (Table 1)

CDCA increased the total bile acid content of plasma and faeces and decreased hepatic *Cyp7a1* mRNA. CDCA reduced body weight but not liver weight or glycogen content. CDCA tended to reduce hepatic triglyceride content (*p* = 0.06), and it reduced plasma glucose and insulin and increased plasma HDL cholesterol. CDCA-treated animals had higher serum ALT *vs.* controls but exhibited no change in ALP or albumin.

### Effects of CDCA on glucocorticoid metabolism

**Fig. 3:**
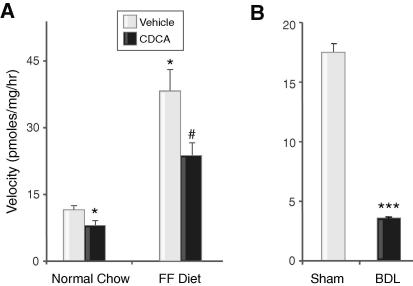
***In vivo*elevation of bile acids inhibits hepatic 5β-reductase in rats**. (A) Hepatic 5β-reductase activity was inhibited in rats following dietary CDCA (filled) *vs.* Control (open) and (B) following BDL (filled) *vs.* sham operation (open). Mean ± SEM; ^∗^*p* <0.05 *vs.* Control, ^#^*p* <0.05 *vs.* Fat-free (FF) diet, ^∗∗∗^*p* <0.001 *vs.* Sham.

Hepatic 5β-reductase activity was reduced by CDCA, using 25 nM substrate (Fig. 3A); this inhibition was overcome by substrate concentrations of 1 μM (0.61 ± 0.14 Control *vs.* 0.56 ± 0.10 CDCA nmol/mg/h). Activity did not correlate with LFTs (all *p* >0.10, *r* < ± 0.39). CDCA did not alter 3αHSD activity or the abundance of mRNAs encoding metabolising enzymes (Table 1).

### Effects of CDCA on the HPA axis and glucocorticoid action (Table 1)

**Fig. 4:**
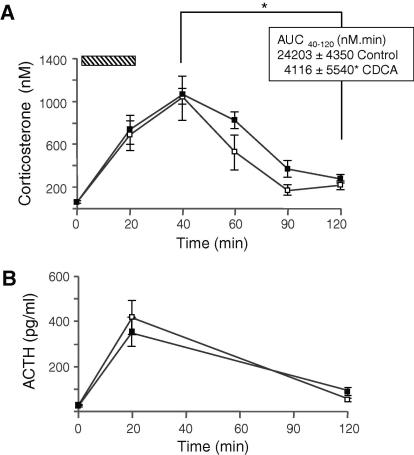
**Dietary CDCA delays recovery from acute stress in rats**. Following restraint (hatched), animals treated with CDCA (filled) had a delay in the return to basal levels of corticosterone (A) but not ACTH (B), compared to Controls (open). Mean ± SEM; ^∗^*p* <0.05 *vs.* Control.

CDCA reduced the excretion of 5β-THB, adrenal weight and adrenal *Cyp11b1* (11β-hydroxylase) gene transcription. Baseline plasma corticosterone levels were comparable between groups. Following acute restraint, CDCA-treated animals had a delay in the return to basal corticosterone levels (Fig. 4).

### Effects of removing bile acids with a fat-free diet and CDCA supplementation in rats (Table 1)

An FF diet was administered to reduce hepatic bile acid concentration; bile acid production rates were reduced (indicated by faecal bile acids), but circulating levels were normal. The FF diet was supplemented with CDCA (FF/CDCA) to test which effects of the FF diet were attributable to bile acid deficiency. Supplementation suppressed hepatic *Cyp7a1* and increased circulating and faecal levels of bile acids.

The FF diet reduced liver weight, circulating glucose and insulin and increased circulating triglycerides and NEFAs compared to control. CDCA replacement reduced weight gain, decreased plasma insulin and liver triglycerides and tended to decrease glucose (*p* = 0.07), but it increased total and HDL-cholesterol compared to the FF diet alone. The FF diet did not affect liver function compared to controls; however, CDCA supplementation in FF animals increased ALT but not ALP or albumin.

### Effects of FF ± CDCA on glucocorticoid-metabolising enzymes

5β-Reductase activity was greater on the FF diet than on chow, while CDCA supplementation on an FF diet reduced 5β-reductase activity when assayed with a substrate concentration of 25 nM (Fig. 3A) but not 1 μM (0.51 ± 0.05 FF *vs.* 0.45 ± 0.10 FF/CDCA nmol/mg/h). The activities of 5β-reductase did not correlate with LFTs (all *p* >0.20, *r* < ±0.35). There were no differences between groups in the activity of 3αHSD or in the levels of mRNAs for metabolising enzymes.

### Effects of FF ± CDCA on glucocorticoid action and the HPA axis

FF diet did not affect urinary corticosterone metabolites compared to Control animals. However, CDCA supplementation reduced urinary total and 5β-reduced corticosterone metabolites and also lowered the circulating concentration of corticosterone (Table 1) compared to FF animals. The abundance of adrenal *Cyp11b1* mRNA was suppressed by CDCA.

### Effects of BDL on steroid metabolism and synthesis in rats

Decompensated liver cirrhosis following BDL was confirmed [13] and was associated with elevated serum bile acids (111 ± 16; *p* <0.05 *vs.* 22 ± 1.8 μM) compared to controls. Plasma corticosterone was not altered by BDL (144 ± 39.6 *vs.* 112 ± 18.5 nM), and adrenal weight was unchanged, as reported previously [13]. *Ex vivo* metabolism of corticosterone by 5β-reductase was significantly impaired, with a substrate concentration of 25 nM (Fig. 3B). This inhibition was overcome only partially using higher (1 μM) concentrations of substrate (1.20 ± 0.3; *p* <0.05 *vs.* 4.32 ± 0.35 nmol/mg/h). Abundances of mRNAs for hepatic 5β-reductase and 3αHSD (Table 2) were reduced in the absence of changes in *Cyp7a1* or 18S. Adrenal *Cyp11b1* mRNA abundance was reduced following BDL.

### Effects of acute cholestasis on cortisol metabolism in humans (Table 3)

Women with obstructive jaundice had elevated serum bilirubin (142 ± 43 μM), ALT (287 ± 57 IU/L), ALP (500 ± 109 IU/L), γ-glutamyl transferase (686 ± 213 IU/L), and bile acid (276 ± 51 μM) concentrations, and the diagnosis of gallstone disease was confirmed at ERCP. Compared to controls, absolute excretion of the major urinary cortisol metabolites was not different relative to urinary creatinine, but those with obstructive jaundice excreted a relatively lower proportion of total cortisol metabolites as 5β-tetrahydrocortisol and had a less significant reduction in relative 5α-tetrahydrocortisol excretion.

## Discussion

This study shows that bile acids, particularly CDCA, are potent competitive and transcriptional inhibitors of rat hepatic 5β-reductase *in vitro*. Moreover, CDCA represses the activity of the human 5β-reductase promoter. These effects were confirmed to be of physiological relevance in rat models *in vivo*. Manipulations predicting increased hepatic bile acids caused impaired rates of 5β-reduction of glucocorticoids in tissue homogenates, which were overcome *ex vivo* with excess substrate, suggesting competitive inhibition. In contrast, 5β-reductase activity was increased by feeding rats an FF diet (which lowered bile acid concentrations), and this effect was reversed by CDCA supplementation. These effects of bile acids also appear to be important in liver disease. In rats with cholestasis, 5β-reductase activity and transcription were reduced, while in humans with biliary obstruction by gallstones, there was a lower relative urinary excretion of 5β-reduced cortisol metabolites. In several of these models, inhibition of 5β-reductase was accompanied by evidence of down-regulation of the HPA axis, with reduced total daily production rates of glucocorticoids, lower production of 3α,5β-reduced metabolites, and a reduction in adrenal weight and *Cyp11b1* expression.

*In vitro* bile acids act as competitive inhibitors of enzymes in the bile acid synthetic cascade [9], and they can also influence the transcription of genes, often via interactions with FXR. Although bile acids have been shown to inhibit 5β-reduction of aldosterone *in vitro* [17], this study is the first to show an inhibition of 5β-reduction of glucocorticoids both *in vitro* and *in vivo*. CDCA, along with its conjugates, was the most potent bile acid tested, with a *K*_i_ similar to its endogenous concentrations in the enterohepatic circulation [9]. The effect was mediated through competitive inhibition of the rate-determining enzyme, 5β-reductase, and not via alterations in 3αHSD activity. CDCA resembles many steroidal substrates for 5β-reductase, having a 3α,5β configuration, and thus may interact with the catalytic site [29]. The difference in potency between CDCA and other bile acids suggests that the interactions with 5β-reductase are independent of their detergent-like properties, but it may indicate that the greater hydrophobicity of CDCA encourages interactions with or without penetration into the active site. Although CA is the most abundant bile acid in healthy rodents, CDCA becomes more abundant in disease [30]. In cultured hepatoma cells, high concentrations of CDCA also reduced the transcript abundance of only 5β-reductase and suppressed its promoter activity in transfection studies. Preliminary examination of the 5′ flanking region of the 5β-reductase gene revealed an FXR-RXR consensus sequence at +18 to +30, but functional activity of this site was not confirmed here. Of note, 5β-reductase has been identified as an FXR-responsive gene by microarray [31].

Following short-term dietary manipulations in rats, inhibition of 5β-reductase activity occurred *in vivo* in the absence of changes in abundance of its mRNA or protein. The similar expression levels may be explained by the inhibition of 5β-reductase activity being competed away by excess substrate *ex vivo*. In other words, at the concentrations of bile acids achieved, it is unlikely that the suppression of gene transcription observed *in vitro* explains the reduction in 5β-reduced steroids *in vivo*. Dietary administration of CDCA not only caused an increase in hepatic and circulatory bile acid concentrations but also altered liver function, inducing mild inflammation [32] without cholestasis. This might be a confounding effect, but, reassuringly, the inhibition of glucocorticoid metabolism by CDCA was also observed on an FF-diet background [18], where hepatic bile acids and bile acid synthesis rates were reduced markedly compared to normal chow, without altering liver function. The consistency of our findings with the FF diet (in which there was no hepatic inflammation) and with CDCA supplementation suggests that the changes in glucocorticoid metabolism are mediated by altered bile acid levels. Moreover, previous studies investigating inflammatory hepatic conditions have demonstrated increased, rather than decreased, 5β-reduction of glucocorticoids [33].

The extent of inhibition of steroid metabolism was more marked in animals following BDL, in which suppression of the HPA axis has been demonstrated [4]. In these animals, cholestasis had developed, and liver function deteriorated. Circulating bile acid concentrations were elevated following BDL. Unlike the dietary manipulations, BDL resulted in altered mRNA levels for hepatic A-ring reductases, and reduced 5β-reductase expression is supported by the inability of high concentrations of substrate to fully overcome the inhibition of glucocorticoid metabolism in this model. This suggests that, unlike the competitive inhibition of steroid-metabolising enzymes, the transcriptional effects of bile acids are more important with the most extreme variations in bile acid concentrations. This finding was specific to enzymes involved with steroid metabolism, not affecting *Cyp7a1* [30] or 18S transcription, but it may reflect a difference in the nature of the cellular composition of the liver, once fibrotic damage commences.

To our knowledge, this is the first study to demonstrate the consequences *in vivo* of alterations in hepatic glucocorticoid metabolism by bile acids on HPA axis regulation. Basal circulating concentrations of corticosterone were not altered with bile acid treatment. However, suppression of the HPA axis was apparent in the reductions in both adrenal size and 11β-hydroxylase gene transcripts, as well as in the marked suppression in the daily production rates of glucocorticoids, resulting in reduced amounts of urinary steroids. The dynamic response of the HPA axis to restraint stress was also different following treatment. Animals receiving dietary CDCA were still able to mount a healthy peak response, unlike cholestatic rats [4], perhaps due to the lesser severity and duration of the insult. However, following dietary CDCA, there was a significant delay in the rate at which circulating corticosterone levels normalised, consistent with impaired rates of clearance of the steroids by 5β-reduction. The impact of CDCA on adrenal gland size was less evident on the FF diet; however, the feedback control of the HPA axis may have been altered due to a non-specific effect of the high sucrose content of the FF diet [34].

In patients who have been hospitalised, cortisol secretion is usually demonstrably increased, especially at night. However, consistent with down-regulation of the HPA axis, we did not find elevated absolute excretion rates of cortisol metabolites in women admitted to the hospital with common bile duct obstruction. We did find, however, that relative excretion of the major 5β-reduced cortisol metabolite, 5β-tetrahydrocortisol, was decreased. It appears that 5β-reductase is not the only enzyme disrupted in these patients; excretion of 5α-reduced cortisol metabolites was also relatively low. Interestingly, although bile acids are known to inhibit both isozymes of 11βHSD [8,14–16], and inhibition of renal 11βHSD2 has been invoked as an explanation for sodium retention in patients with liver disease, the ratio of cortisol to cortisone metabolites was strikingly altered in favour of cortisone metabolites in our patients. This suggests that inhibition of A-ring reduction of cortisol and/or of 11βHSD1 is more important than inhibition of renal 11βHSD2 (which would elevate the cortisol/cortisone metabolite ratio). Moreover, as illustrated in the rare condition of cortisone reductase deficiency [35], loss of 11βHSD1 is associated with impaired regeneration of cortisol from cortisone and a compensatory increase in HPA axis activity. Because our patients exhibited a paradoxical failure to increase cortisol secretion, we suggest that the inhibition of A-ring reductases, and hence suppression of the HPA axis, is the dominant effect. 5β-Reductase expression is suppressed by androgens [36], and thus the inhibitory effects of bile acids may be more obvious in women (in whom obstructive biliary disease is more prevalent [37]).

In summary, the data presented here suggest an important and novel role for bile acids in regulating the pattern and consequences of glucocorticoid metabolism within the liver. The elevated levels of bile acids arising during cholestasis may contribute to the down-regulation of the HPA axis and hence the apparent adrenal insufficiency associated with liver disease. Therefore, sequestration of bile acids may be beneficial at early stages of this disease, not only to improve pruritis but also perhaps to improve responsiveness to stress.

## Figures and Tables

**Table 1:** Physiological parameters following dietary manipulation.

	**Control**	**CDCA**	**Fat-free**	**Fat-free/CDCA**
BW change (g)	161.4 ± 7.1	121.9 ± 7.0∗	197.1 ± 6.6∗	174.3 ± 4.5^#^
Adrenal weight (mg/g BW)	0.082 ± 0.005	0.066 ± 0.005∗	0.11 ± 0.003∗	0.11 ± 0.003
*Cyp11b1* copies mRNA/μg total RNA	9.13 ± 0.90 × 10^7^	7.01 ± 0.74 × 10^7^∗	8.14 ± 1.59 × 10^7^	4.40 ± 0.43 × 10^7#^

**Liver**				
Weight (mg/g BW)	45.3 ± 0.9	43.7 ± 1.06	38.2 ± 3.0∗	33.3 ± 3.1
Glycogen (μmol/mg)	0.71 ± 0.15	0.68 ± 0.14	0.93 ± 0.09	1.07 ± 0.19
Triglycerides (mg/g)	14.7 ± 1.8	10.4 ± 1.5	18.6 ± 2.1	11.2 ± 1.6^#^
ALT (IU/L)	78 ± 6.5	211 ± 31.8∗	36 ± 2.4	343 ± 118^#^
ALP (IU/L)	9.25 ± 3.9	30 ± 17.7	4 ± 0.6	5 ± 1.8
Albumin (g/L)	39 ± 1.3	41 ± 3.0	42 ± 1.3	40 ± 0.6
5β-Reductase mRNA/18S	0.79 ± 0.15	0.62 ± 0.04	0.42 ± 0.07	0.35 ± 0.04
3αHSD mRNA/18S	0.67 ± 0.18	1.02 ± 0.27	0.44 ± 0.75	0.61 ± 0.25
3αHSD activity (nmol/mg/hr)	6.22 ± 1.1	6.22 ± 0.6	9.55 ± 1.6	9.35 ± 2.1

**Plasma**				
Corticosterone (nM)	78 ± 19	82 ± 26	85 ± 10	53 ± 7^#^
Glucose (mM)	8.4 ± 0.7	5.4 ± 0.7∗	5.4 ± 0.6∗	4.2 ± 0.5
Insulin (μg/L)	4.74 ± 0.60	2.87 ± 0.58∗	3.05 ± 0.48∗	1.78 ± 0.19^#^
Triglycerides (mM)	1.5 ± 0.2	1.3 ± 0.1	3.1 ± 0.3∗	1.3 ± 0.2^#^
Cholesterol (mM)	1.89 ± 0.09	2.18 ± 0.11	1.81 ± 0.12	2.4 ± 0.1^#^
HDL cholesterol (mM)	1.33 ± 0.08	1.59 ± 0.09∗	1.1 ± 0.1	1.6 ± 0.08^#^
NEFA (mM)	0.25 ± 0.03	0.27 ± 0.03	0.43 ± 0.04∗	0.49 ± 0.06

**Bile acids**				
*Cyp7a1* mRNA/U1	1.36 ± 0.06	0.70 ± 0.03∗	4.50 ± 0.56	2.78 ± 0.57^#^
Plasma (μM)	28.0 ± 8.7	73.6 ± 15.9∗	31.9 ± 6.2	108.03 ± 16.4^#^
Hepatic (nmol/g)	84.3 ± 17.7	115.7 ± 16.4	25.1 ± 7.1∗	34.8 ± 3.1
Faecal (μmol/day)	7.0 ± 0.8	12.5 ± 2.1∗	1.0 ± 0.3∗	5.3 ± 1.0^#^

**Urinary steroids (ng/24 hr)**				
5β-THB	377 ± 68	213 ± 45∗	305 ± 26	178 ± 22^#^
5α-THB	132 ± 32	187 ± 39	268 ± 49∗	240 ± 27
Total	508 ± 77	400 ± 47	651 ± 116	392 ± 51^#^


CDCA, chenodeoxycholic acid; BW, body weight; ALT, alanine transaminase; ALP, alkaline phosphatase; *Cyp11b1*, 11β-hydroxylase; NEFA, non-esterified fatty acids; HSD, hydroxysteroid dehydrogenase; *Cyp7a1*, cholesterol 7a-hydroxylase; THB, tetrahydrocorticosterone. Mean ± SEM; ^∗^*p* <0.05 vs Control, ^#^*p* <0.05 vs Fat-free.

**Table 2:** mRNA transcripts of enzymes following BDL.

	**Sham**	**BDL**
**Hepatic**		
5β-Reductase	9.07 ± 6.91	0.85 ± 0.60∗
3αHSD	7.07 ± 3.60	0.55 ± 0.34∗∗
*Cyp7a1*	2.11 ± 0.81	2.33 ± 0.56

**Adrenal**		
*Cyp11b1*	8.25 ± 0.74	5.74 ± 0.76∗

BDL, bile duct ligation; HSD, hydroxysteroid dehydrogenase; *Cyp7a1*, cholesterol 7a-hydroxylase; *Cyp11b1*, 11β-hydroxylase. Hepatic genes corrected for 18S (unchanged between groups). *Cyp11b1* is copy no/μgRNA×10^7^. Mean ± SEM; ^∗^*p* <0.05, ^∗∗^*p* <0.01.

**Table 3:** Urinary cortisol metabolites (μg/mg creatinine) in patients with obstructive jaundice.

	**Obstructive jaundice**	**Control**
*n*	8	5
Cortisol	0.49 ± 0.13	0.11 ± 0.02
5β-THF	2.20 ± 0.89	2.49 ± 0.30
5α-THF	2.29 ± 0.78	2.95 ± 0.35
THE	6.39 ± 1.48	2.99 ± 0.32
5β-THF/5α-THF	1.45 ± 0.68	0.84 ± 0.02
5β-THF/cortisol	5.09 ± 1.36∗∗∗	20.11 ± 2.15
5α-THF/cortisol	8.88 ± 3.49∗	23.77 ± 3.12
(5β-THF + 5α-THF)/THE	0.71 ± 0.15∗∗∗	1.82 ± 0.05

THF, tetrahydrocortisol; THE, tetrahydrocortisone. Mean ± SEM. ^∗^*p* <0.05, ^∗∗∗^*p* <0.001.
